# Development of Biodegradable Chewing Gum Using Plasticized Poly(Lactic Acid)/*Pistacia atlantica* Gum Blend: A Novel Gum Base Elastomer for Sustainable Formulation

**DOI:** 10.1002/fsn3.72322

**Published:** 2026-09-07

**Authors:** Mona Kaveh, Samira Yeganehzad, Mohammad Ali Hesarinejad

**Affiliations:** ^1^ Department of Food Sensory and Cognitive Science Research Institute of Food Science and Technology (RIFST) Mashhad Iran

**Keywords:** eco‐friendly, elastomer, Saqqez gum, sensory evaluation

## Abstract

Environmental concerns necessitate the exploration of novel biodegradable alternatives in chewing gum formulations. This study focused on developing a biodegradable chewing gum based on a plasticized poly(lactic acid)/ *Pistacia atlantica* gum blend, aiming for desirable physical and mechanical properties. In this study, the effects of the proportions of four components, elastomeric biodegradable blend, xylitol, glycerin, and calcium carbonate, on textural and sensory characteristics of chewing gum were evaluated using a 28‐run, four‐component D‐optimal mixture design with constrained formulation ranges. The optimized composition was also characterized and compared with two commercial samples using textural, thermal, morphological, and biodegradability analyses, and quantitative descriptive analysis for sensory evaluation. According to the findings, the biodegradable elastomer blend and xylitol generally enhanced the preference for all sensory attributes, while glycerin reduced the hardness, chewiness, and cohesiveness of the chewing gums. The optimal composition was determined to be 39.40% plasticized poly(lactic acid)/Saqqez gum blend, 30.59% xylitol, 20.00% glycerin, and 10.00% calcium carbonate. Texture analysis revealed that the optimal chewing gum exhibited high cohesiveness, springiness, and chewiness. The optimal sample showed higher melting temperatures, attributed to its lower moisture content compared to the commercial variants. Quantitative descriptive analysis indicated that the intensity of hardness, moistness, and sandiness were relatively similar in both optimal and Saqqez commercial chewing gum, placing them in the same group based on the significance level. Overall, the results suggest that this novel biodegradable chewing gum holds significant promise for developing highly beneficial products in the food industry, with potential for both environmental sustainability and health benefits.

## Graphical abstract

Eco‐friendly Chewing Gum: A Novel Formulation Based on Biodegradable PLA/ *Pistacia atlantica* Blend.

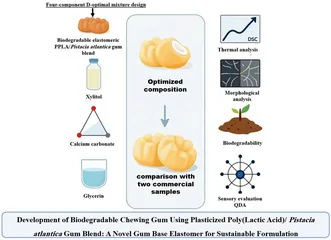

## 1 Introduction

According to the National Consumer Survey, approximately 56% of U.S. households consume chewing gum, with an average consumption of 160 to 180 sticks per person annually. This equates to an annual production of 1.74 trillion gum sticks (Kaveh et al. 2023). Consequently, most commercial chewing gums are non‐degradable due to the presence of petroleum‐based compounds in their gum base, which raises significant environmental concerns (Kaveh et al. 2023). On the other hand, research on wax‐free chewing gum bases (Synosky and Reed 1994) has been conducted due to their classification as low‐calorie products (McGrew and Synosky 1994). Additionally, some studies have demonstrated that sugar‐free chewing gums containing xylitol and other polyols have direct anticaries effects, including the remineralization of insipient lesions (Hanham and Addy 2001; Ingle et al. 2013). Ongoing efforts aim to replace these compounds with natural, biodegradable and health‐oriented alternatives. Recent advances in biopolymer systems, particularly PVA‐based biocomposites, have demonstrated notable improvements in mechanical and functional properties through the incorporation of natural fillers and bio‐based modifiers (Ghozali et al. 2023).

Chewing gum is a complex mixture characterized by its elastic, flexible, and expandable properties. It consists of two main phases: a water‐insoluble gum base and a water‐soluble component (Kaveh et al. 2023; Thivya et al. 2021). The gum base, which does not dissolve in the oral cavity or saliva during chewing, typically constitutes < 50% of the final product and includes elastomers, resins, plasticizers, fillers, emulsifiers, and antioxidants. This gum base significantly influences overall acceptability and determines the ultimate chewing gum textural and sensory properties. These ingredients exhibit high compatibility and do not chemically react with each other (Estruch 2008). The water‐soluble phase in chewing gums generally comprises sweeteners and emulsifiers. During chewing, some water‐soluble components are released through saliva, while others volatilize in the oral cavity, affecting organoleptic properties. Interactions among these components impact the texture and sensory quality of the chewing gum. Interestingly, the specific formulation of chewing gum remains a closely guarded secret for most manufacturers (Tisdale and Wilkins 2014).

Developing an innovative product requires a detailed presentation of their formulation, including components, characteristics, and limitations (Galvan et al. 2021). Food scientists meticulously investigate and optimize mixtures to achieve desired textural and sensory traits in the final product. Experimental designs for mixtures serve as valuable tools for analyzing the impact of various components in formulations (Squeo et al. 2021). Among the various experimental designs, mixture designs are predominant (Galvan et al. 2021), which facilitate the determination of optimal conditions for product formulations or industrial processes. By employing predictive equations and mathematical algorithms, mixture designs help identify the best ratio of main components. These models aim to achieve high‐quality outcomes, minimize costs, optimize single or multiple responses simultaneously, and address constraints (Das et al. 2016; Orives et al. 2014).

Saqqez gum, derived from *Pistacia atlantica* trees in the Zagros zone of Iran, served as a non‐toxic, renewable, and biodegradable oleo‐gum resin, which generally includes polysaccharides, α‐pinene, terpenoids, and flavonoids (Kamrani et al. 2007). Saqqez gum, a semi‐dense, adhesive, and liquid resin, has both industrial and traditional applications, particularly in chewing gum. Historically, it has been recognized for its valuable pharmaceutical, dental, and oral health properties. Saqqez gum offers benefits such as breath freshening, antiseptic properties, and relief from gastrointestinal issues and motion sickness. Additionally, it can inhibit the growth and acid production of microorganisms responsible for dental caries (Rahbar Saadat et al. 2016). In our earlier study (Kaveh et al. 2024), we successfully blended Saqqez gum and plasticized poly(lactic acid) (PPLA) with 16% acetyl‐tributyl‐citrate, resulting in flexible PPLA/Saqqez gum blend.

In our current study, we developed a novel free‐sugar, free‐wax, and biodegradable chewing gum using our flexible elastomeric compound. The aim of this study was to investigate the impact of four main components (gum base elastomer, bulking sweetener, emulsifier, and filler) on the textural and sensory characteristics of the chewing gum using a D‐optimal mixture design. Additionally, to characterize and compare the optimal composition with two commercial samples, textural, thermal, morphological, biodegradability analyses, along with quantitative descriptive analysis for sensory evaluation, was conducted. This study emphasizes the potential for developing environmentally friendly and healthy chewing gum.

## 2 Materials and Methods

### 2.1 Materials

The Saqqez gum from Bane tree ( *Pistacia atlantica* subsp. mutica) was sourced from Saqqez production Van Co. (Kurdestan, Iran). Commercial poly(lactic acid) (PLA) pellets were provided from NatureWorks (IngeoTM Biopolymer, 4043D). Acetyl‐tributyl‐citrate was purchased from Sigma‐Aldrich Co., while JONCRYL ADR‐4368‐C was supplied by BASF in Germany. Xylitol was purchased from Roquette (France). Calcium carbonate was prepared from Merck (CAS 471‐34‐1). Glycerin was sourced from Wanco (Malaysia). All other chemicals were of analytical grade and purchased from Merck (Darmstadt, Germany) and/or Sigma–Aldrich Co. (USA).

### 2.2 Experimental Design and Statistical Analysis for Optimization

The effect of formulation component ratios on the textural and sensorial characteristics of chewing gum was assessed using a D‐optimal mixture design. This approach allows for the efficient exploration of the formulation space, especially when constraints are present. The experimental design was developed and analyzed using Design‐Expert software (v.7.6.1; Stat‐Ease Inc., Minneapolis, MN, USA). A 28‐run experiment was designed for a four‐component mixture system with multiple constraints on the component ratios. The independent variables were: plasticized Poly(Lactic Acid)/ *Pistacia atlantica* gum blend (PPLA/Saqqez gum, *X1*), xylitol (*X2*), glycerol (*X3*), and calcium carbonate (*X4*). The minimum and maximum levels for each component were defined to ensure a feasible and practical formulation space, as detailed in Table 1. Textural attributes (hardness, cohesiveness, springiness, chewiness, rupture point) and sensory characteristics (texture, appearance, odor, color, overall acceptance) were evaluated as dependent variables. The 28 experimental runs designed within this study are detailed in Table 2.

**TABLE 1:** Independent variables, their constraints and function in chewing gum, and dependent variables, their definition, and considered target of the D‐optimal mixture design.

Independent variables	Function in chewing gum	Constraint levels (% w/w)
PPLA/Saqqez gum (*X1*)	Elastomer	10–40
Xylitol (*X2*)	Bulking sweeteners	30–70
Glycerol (*X3*)	Emulsifier/plasticizer	20–40
Calcium carbonate (*X4*)	Filler	10–40

Dependent variables	Definition	Considered target
Hardness	The resistance of the sample to deformation	Minimize
Cohesiveness	The ratio of positive force area during the second compression to that during the first compression	Maximize
Springiness	The rate at which a deformed material returns to its original state after the removal of the deforming force	Maximize
Chewiness	Hardness × Cohesiveness × Springiness	Maximize
Rupture point	Value of the bending force at a starting point of a macroscopic failure of the material due to the significant deformation thereof	Minimize
Texture	The general textural properties of samples that perceived by chewing such as hardness, chewiness, and stickiness	Maximize
Appearance	The sensations perceived by sight and touch	Maximize
Odor	The sensations perceived by smell	Maximize
Color	Visual perception of samples' appearance color	Maximize
Overall acceptance	How well samples are liked or preferred by consumers	Maximize

**TABLE 2:** Proportion of 4‐components in the formulation of chewing gums based on mixture design (g/100 g).

Samples	PPLA/Saqqez	Xylitol	Glycerin	Calcium carbonate
1	40	30	20	10
2	26.67	30	33.33	10
3	26.67	30	33.33	10
4	10	30	33.33	26.67
5	10	43.33	26.67	20
6	10	60	20	10
7	16.67	36.67	30	16.67
8	10	60	20	10
9	10	45	20	25
10	13.33	33.33	25	28.33
11	25	45	20	10
12	10	50	30	10
13	16.67	36.67	30	16.67
14	10	30	20	40
15	10	30	40	20
16	28.33	33.33	25	13.33
17	16.67	36.67	30	16.67
18	16.67	36.67	30	16.67
19	10	45	20	25
20	20	40	20	20
21	25	45	20	10
22	10	40	40	10
23	25	30	20	25
24	23.33	30	26.67	20
25	25	30	20	25
26	20	30	40	10
27	40	30	20	10
28	10	30	20	40

After collecting data from each response, statistical analysis was performed using appropriate models (linear, quadratic and special cubic, depending on the degree of fit, predictive power and robustness of the model) to model the responses using Design Expert software. Coefficient values were provided for the selected models. Dependent variables, such as texture and sensory properties of samples, were analyzed. The models were subjected to analysis of variance (ANOVA) to determine their significance (*p* < 0.05), *R* ^2^ values, and lack of fit. Significant dependent variables were further analyzed using the software's statistical tool. Based on this analysis, polynomial equations (linear and/or quadratic) correlating the independent variables with each response were constructed and used in subsequent analysis. The coefficients for *X1*–*X4* describe the effect of these variables on the responses, where a larger coefficient indicates a more potent impact of the factor on the response. The D‐optimal mixture design developed a model to predict the impact of formulation components. The predicted models were then validated. Model adequacy was assessed using several statistical criteria, including Adjusted *R* ^2^ (Adj‐*R* ^2^), Predicted *R* ^2^ (Pred‐*R* ^2^), and the *p*‐values associated with the Lack‐of‐fit test. Models were selected when they exhibited high Adj‐*R* ^2^ and Pred‐*R* ^2^ values (typically differing by < 0.2), and a non‐significant Lack‐of‐fit (*p* > 0.05), indicating a good agreement between the experimental data and the model predictions. The final model selection also considered the overall significance of the model (low *p*‐value for the model itself) and the interpretability of the coefficients.

For optimization, a desirability function approach was utilized. This numerical optimization method facilitates the simultaneous consideration of multiple response variables, each with its own objective (maximization or minimization), to identify a globally optimal solution. The specific targets for each response variable were set as follows: hardness and rupture point were set to be minimized, while cohesiveness, springiness, chewiness, texture, appearance, odor, color, and overall acceptance were set to be maximized. The software, specifically the optimization module, then generated a composite desirability value for each formulation. The final optimum composition was mathematically derived as the specific ingredient combination that yielded the highest calculated composite desirability score, thereby representing the best overall compromise among all targeted responses. To validate these predictions, the models' accuracy was confirmed through statistical criteria (Adj‐*R* ^2^, Pred‐*R* ^2^, Lack‐of‐fit *p* > 0.05). Furthermore, the optimal formulation identified through this process was experimentally prepared, and its properties were evaluated, demonstrating good agreement with the model predictions, thereby validating the optimization procedure and ensuring its reproducibility and evaluability.

Model adequacy was evaluated using Analysis of Variance (ANOVA). For all response variables, a quadratic mixture model was determined to be the best fit based on statistical significance (*p* < 0.05), lack‐of‐fit tests (*p* > 0.05), and the highest Adequate Precision values (all > 4.0), indicating that the models are significant and provide reliable predictions within the experimental domain. Residual diagnostics, including normal probability plots of residuals and plots of residuals versus predicted values, confirmed the validity of the model assumptions.

### 2.3 Samples Preparation

#### 2.3.1 Production of PPLA/Saqqez Blend as Elastomer

The PLA pellets and Saqqez gum were dried separately overnight in a vacuum oven (Heraeus Vacum therm VT6130P, Germany) at 80°C and 45°C, respectively. To prepare the plasticized PLA (PPLA), 16% acetyl‐tributyl‐citrate was directly melt‐mixed with PLA using an internal mixer (RheoSense, IPPI, Iran) at 160°C and 60 rpm for 15 min, and then the prepared PPLA was subsequently melt‐mixed with Saqqez gum at a 70:30 ratio and JONCRYL (2 phr) as a compatibilizer, in the internal mixer at 150°C and 60 rpm for 10 min (Kaveh et al. 2024).

#### 2.3.2 Production of Chewing Gum Samples

**FIGURE 1:**
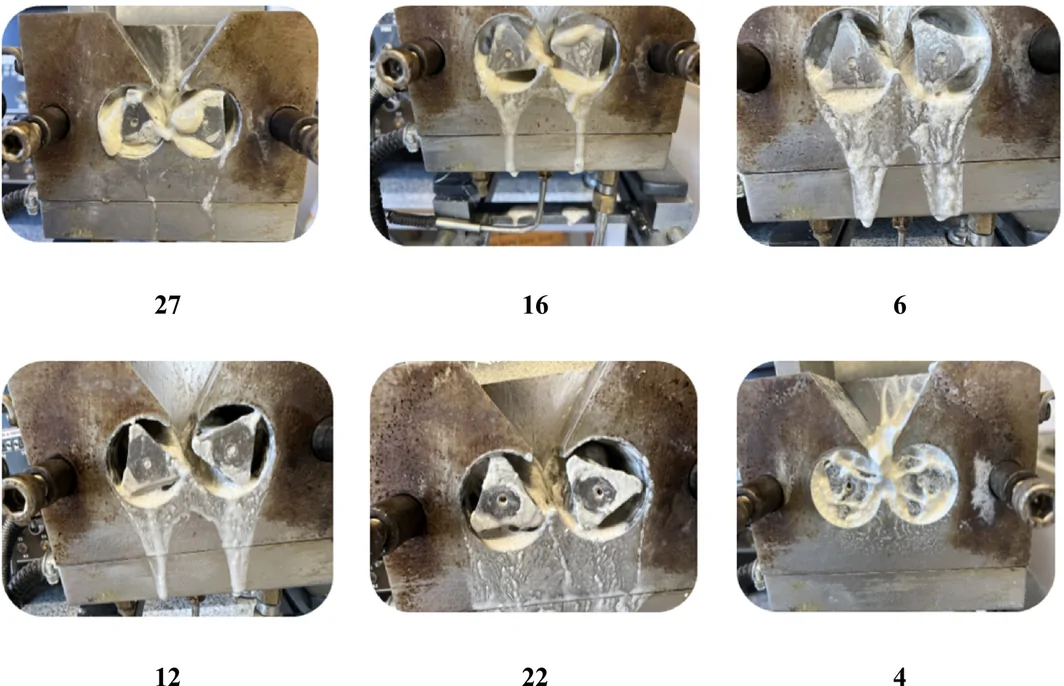
The final appearance of some samples produced before being removed from the internal mixer machine, with each image labeled according to the sample number.

Based on the quantitative formulations of chewing gum samples shown in table 2, the production process followed several steps outlined by Kaveh et al. (2024), with some modification and using an internal mixer equipped with a roller‐type rotor (RheoSense, IPPI, Iran) (Figure 1). Initially, the molten PPLA/Saqqez gum blend, melted at 100°C, was mixed with glycerol, xylitol, and calcium carbonate for 15 min at 80°C. Subsequently, the resulting dough was shaped using a manual roller. The samples were then conditioned at 25°C and 45% relative humidity for 1 week to harden. Finally, the samples were packed in nylon covers and stored in a dry and cool place until testing (Kaveh et al. 2024).

### 2.4 Optimization of Chewing Gum Samples

For the optimization step, the following three tests were considered:

#### 2.4.1 Textural Analyses

Oval‐shaped chewing gum samples were prepared with a major axis diameter of 2 cm, a minor axis diameter of 1.5 cm, and a thickness of 4 mm. Each sample had a mass of approximately 2.0 g. the texture profile analysis (TPA) method was adapted from Kaveh et al. (2024) and Mohammadi et al. (2019) with modifications. TPA was conducted using a TA‐XT Plus texture analyzer machine (Stable Micro Systems, Godalming, UK) equipped with a cylindrical probe P/20 (20 mm in height, 20 mm in diameter). Samples were compressed twice to 60% of their original height at a crosshead speed of 5 mm/s. All samples were carried out at room temperature. The evaluated parameters included hardness, cohesiveness, springiness, and chewiness.

Additionally, the cutting performance of the chewing gum samples was evaluated using a Stable Micro System Model TA. XT2i texture analysis machine (Stable Micro Systems, Godalming, UK). The cutting test was carried out with a craft knife blade (HDP/BS, Batch No. 13,155) at speeds of 1 mm/s for pre‐test compression, 2 mm/s during the cut, and 1 mm/s for post‐test, respectively (Saberi et al. 2018).

No chewing or simulated chewing was performed on samples in the current study; all analyses were carried out on unchewed samples to ensure instrumental standardization.

#### 2.4.2 Consumer Acceptance Sensory Evaluation

An acceptance test utilizing a 5‐point hedonic scale (1 = I don't like it at all, 2 = I don't like it, 3 = I neither like nor dislike it, 4 = I like it, 5 = I like it very much) was conducted to evaluate various attributes, including texture, appearance, odor, color, and overall acceptance. The test involved 50 regular consumers of chewing gum, aged between 18 and 50 years, comprising 56% women and 44% men. The experiment was designed as a completely balanced block design, and the results were analyzed with a significance level of (*p* < 0.05) (Maleki et al. 2019).

### 2.5 Characterization of Optimized Sample

In this section, the optimized (Op) chewing gum was characterized and compared with two commercially available chewing gums: the Saqqez commercial (SaC) and a synthetic gum base commercial (SyC) chewing gums. The identities and general characteristics of these commercial samples are detailed and summarized in Table 3. Various tests were conducted, which are detailed as follows:

**TABLE 3:** Characterization of commercial chewing gum samples.

Features	SaC	SyC
Manufacturer	Van Company (Iran)	Biodent Company (Iran)
Gum Base Type	Natural Resin (Saqqez gum)	Synthetic gum base
Primary Sweetener	Xylitol	Xylitol
Key Components	Saqqez gum, Xylitol	Synthetic Base, Xylitol, Flavors
Physical Form	Pellet	Pellet

#### 2.5.1 Moisture and Total Ash Content

The moisture content of the samples was assessed using AOAC 46.950 method. The total ash content was measured in accordance with AOAC method 923.03 (Thiex 2009).

#### 2.5.2 Thermal Properties

Thermal analysis of the Op chewing gum sample, as well as SaC and SyC chewing gums, was conducted by a differential scanning calorimeter (DSC SL800, SPICO, China). The analysis involved determining the glass transition temperature (Tg) and melting temperature (Tm). Samples weighing approximately 15 mg were placed in sample pans, sealed, and introduced into the differential scanning calorimetry (DSC) heating cell. The samples were then heated from 25°C to 250°C at a rate of 5 K/min under a nitrogen atmosphere with a flow rate of 10 mL/min (Mardani et al. 2022).

#### 2.5.3 Morphological Properties

The microstructure and fracture surface morphology of the samples were examined using field emission scanning electron microscopy (FESEM) at an accelerating voltage of 10 kV. The analysis was performed on surfaces that were vacuum‐coated with gold. The FESEM utilized was a TESCAN model MIRA3 from the Czech Republic.

#### 2.5.4 Aerobic Biodegradation

The aerobic biodegradability of the samples was assessed in accordance with ASTM D5988‐18 (2018). This method evaluates the level and rate of aerobic biodegradation of the samples compared to a standard material in contact with soil under controlled laboratory conditions. The biodegradability was determined by measuring the amount of carbon dioxide produced from the biological deterioration of the samples by microorganisms within the temperature range of 20°C–28°C. To ensure microbial diversity, a blend of three soil types (agricultural, forest, and pasture soil) along with a small amount of vegetable compost was used as the matrix soil, following the standard protocol. One gram of Op chewing gum sample powder was utilized as the test sample, while 1 g each of SaC and SyC chewing gum powders. The evaluation was performed on all samples with three repetitions (Moghaddam et al. 2023).

#### 2.5.5 Quantitative Sensory Descriptive Analysis

In this research, six textural characteristics including hardness, chewiness, stickiness, cohesiveness, moistness, and sandiness of three samples were evaluated and analyzed. The quantitative sensory descriptive analysis method, as described by Kiumarsi et al. (2020), was employed to characterize the sensory attributes of the samples through three steps: selecting prospective assessors, training them, and testing the products. Nineteen participants, aged between 20 and 50 years, who were regular chewing gum consumers, were selected and trained. The training aimed to enhance the panel members' sensitivity to the characteristics and to familiarize them with the definitions of each attribute, as well as to improve their differentiation, reproducibility, and evaluation skills. The participants rated the intensity of each of the predefined characteristics on a 5‐point structured scale, where 0 = not perceived and 5 = strong (Kiumarsi et al. 2020), whose definitions and measurement scales are given in Table 4.

**TABLE 4:** Definitions and measurement scales for quantitative sensory descriptive analysis.

Attributes	Definitions	Measurement scales
Hardness	The force required to compress a material between the teeth	0: Cream 5: Fruit candy
Chewiness	Creating the proper consistency and firmness of the sample in the mouth	0: Cotton candy 5: Toffee
Moistness	Describes the watery state of a product	0: Oat biscuit 5: Watermelon
Stickiness	Amount of adhesion to teeth and palate	0: Cooked rice 5: Cheese curls
Cohesiveness	Holding sample components together coherently	0: Cookie 5: Mozzarella cheese
Sandiness	Perceiving the feeling of sand in the mouth	0: Butter 5: Crunchy peanut butter

The intensity values of each attribute were recorded after 5 min. These values were then subjected to analysis of variance (ANOVA), and spider web plots were generated using Minitab software version 16. ANOVA was utilized to analyze differences between samples based on specific characteristics, while spider web plots were employed to describe the sensory attributes of the samples.

#### 2.5.6 Textural Characteristics

TPA test was conducted on the selected samples using the same method as in the Section 2.4.1.

#### 2.5.7 Statistical Analysis for Characterization

All experiments and analyses were conducted in triplicate. The data were subjected to one‐way analysis of variance (ANOVA) and Tukey's test to compare mean values and identify significant differences at a 95% confidence level (*p* < 0.05), using Minitab 16 software.

#### 2.5.8 Ethics/Consent Statement and Approval

The procedure for this study was approved by the Ethics committee of Ferdowsi university of Mashhad, Iran for Scientific Research (Reference no. IR.UM.REC.1404.494) and prior to the experiment, all participants voluntarily signed an informed consent form.

## 3 Results and Discussion

### 3.1 Experimental Design

The response variables obtained from experiments involving 10 different attributes, determination coefficient (*R* ^2^) values, and ANOVA tables for all parameters are presented in Tables S1–S2. All attributes were best modeled by a quadratic polynomial. For the attributes such as hardness, chewiness, rupture point, texture, odor, and color, both the linear and the quadratic models demonstrated statistical significance, indicating their adequacy in describing these responses. Conversely, for attributes like cohesiveness, springiness, appearance, and overall acceptance, only the quadratic model was statistically significant (*p* < 0.05). In these attributes the linear model is inadequate, as shown by low *R* ^2^ values, and the quadratic model offers a significantly better fit. *R* ^2^ measures how well independent variables in statistical models explain the variation in dependent variables (Lewis‐Beck and Skalaban 1990). Notably, most attributes in the quadratic model achieved an *R* ^2^ of approximately 0.9, indicating an excellent fit, especially for all sensory attributes. To avoid overfitting in models with multiple independent variables and the complexity of the model, attention to adjusted *R* ^2^ is necessary. Adjusted *R* ^2^ takes into account the number of predictors (independent variables) and balances model fit with complexity (Bhandari 2020). Higher adjusted *R* ^2^ values suggest a better balance between explaining variation in the dependent variable and avoiding unnecessary complexity in the model. According to the results, the majority of the adjusted *R* ^2^ values exceeded 0.8, which is considered acceptable. With respect to the predicted *R* ^2^ values, it seems that most of the quadratic models exhibit suitable predictive capability for future observations. The special cubic model appears to be aliased or inapplicable for all attributes, as indicated by a standard deviation of 0 and a PRESS value of “+”.

As mentioned earlier, to understand the relationship between the responses and the components, statistical models are fitted to the data. These models are then analyzed using regression models to identify which components significantly influence the mixture's properties. Table S2 presents the findings of the analysis of variance (ANOVA) for quadratic models fitted to various responses of the mixture. The ANOVA table indicates that only the quadratic model is significant, meaning it effectively explains the variability in its respective traits.

According to the ANOVA results, all attributes are significantly influenced by the quadratic model. This suggests that the attributes are affected by the interaction between two or more mixture components. The *F*‐values for appearance, texture, color, and overall acceptance are significantly higher than those for other attributes. Several factors contribute to this issue. Firstly, the quadratic models for these attributes explain a substantial portion of the variance in the data. Secondly, the components in these quadratic models are likely highly significant, enhancing the model's ability to explain the variance. Additionally, the nature of the data related to sensorial attributes, such as appearance, texture, color, and overall acceptance, often exhibits consistent patterns with less noise or variability due to human perception. This consistency facilitates the model's detection of significant effects, leading to low *p*‐values. In summary, the very low *p*‐values for these attributes indicate highly significant models that explain a substantial portion of the variance, making the observed relationships unlikely to be due to chance or random variation. Generally, all independent variables had a significant effect (*p*‐value < 0.05) on the responses.

### 3.2 Effect of Chewing Gum Components on the Responses

In mixture design involving components, polynomial models are used to evaluate the effect of each component on the response variables. Understanding the impact of each component in the formulation allows manufacturers to achieve the desired functional properties and meet consumer expectations (Kaveh et al. 2023). These models rely on coefficient values, which describe the impact of each component or predictor (*X1*, *X2*, *X3*, *X4*, and their interactions) on different attributes or response variables, while holding all other predictors constant. Specifically, these coefficients indicate how the response variable changes with a one‐unit increase in the component. Higher coefficients signify a more potent factor effect on the response. Positive coefficient values suggest a synergistic effect, while negative values indicate an antagonistic influence on the response (Al Hagbani et al. 2018).

Table S3 presents the coefficients for such polynomial models, showing that components *X1*, *X2*, *X3*, and *X4* had significant effects on various attributes. Below are the quadratic mixture regression equations for all significant responses, derived directly from the coefficients in Table S3.

$$\begin{array}{c} Y\;\text{Hardness}=3642.44\;X1+1655.30\;X2-3277.65\;X3+1595.42\;X4\\ \;-1623.63\;X1\;X2+6799.30\;X1\;X3-2399.64\;X1\;X4\\ \;+7118.16\;X2\;X3-544.98\;X2\;X4+7808.67\;X3\;X4 \end{array}$$

$$\begin{array}{c} Y\;\text{Cohesivenes}=0.39\;X1+0.24\;X2-1.26\;X3\\ \;+0.76\;X4+0.09\;X1\;X2+3.67\;X1\;X3\\ \;-1.56\;X1\;X4+1.52\;X2\;X3-1.27\;X2\;X4\\ \;+1.02\;X3\;X4 \end{array}$$

$$\begin{array}{c} Y\;\text{Springiness}=1.00\;X1+1.00\;X2+0.92\;X3+0.99\;X4\\ \;-0.005\;X1\;X2+0.13\;X1\;X3-0.01\;X1\;X4\\ \;+0.10\;X2\;X3+0.02\;X2\;X4+0.13\;X3\;X4 \end{array}$$

$$\begin{array}{c} Y\;\text{Chewiness}=1421.19\;X1+431.59\;X2-4472.36\;X3+1223.43\;X4\\ \;-644.88\;X1\;X2+9965.71\;X1\;X3-3783.14\;X1\;X4\\ \;+5697.29\;X2\;X3-2293.02\;X2\;X4+5630.54\;X3\;X4 \end{array}$$

$$\begin{array}{c} Y\;\text{Rupture}\;p.=7488.24\;X1+2770.63\;X2+92529.28\;X3-13786.76\;X4\\ \;-16800.92\;X1\;X2-1.788\times 10^{5}X1\;X3-37466.81\;X1\;X4\\ \;-94491.03\;X2\;X3+24576.23\;X2\;X4-1.410\times 10^{5}X3\;X4 \end{array}$$

$$\begin{array}{c} Y\;\text{Appearance}=3.55\;X1+3.67\;X2+5.18\;X3+3.43\;X4\\ \;-1.35\;X1\;X2-17.62\;X1\;X3-13.23\;X1\;X4\\ \;+3.58\;X2\;X3+2.11\;X2\;X4-3.45\;X3\;X4 \end{array}$$

$$\begin{array}{c} Y\;\text{Texture}=3.33\;X1+0.26\;X2-0.13\;X3+1.68\;X4+6.95\;X1\;X2\\ \;+6.52\;X1\;X3-8.15\;X1\;X4+1.34\;X2\;X3\\ \;-2.99\;X2\;X4+1.41\;X3\;X4 \end{array}$$

$$\begin{array}{c} \text{YOdor}=4.59X1+4.82X2+4.51\;X3+4.91\;X4-0.63\;X1X2\\ \;-1.60\;X1X3-0.30X1X4+0.14X2X3-1.74X2X4\\ \;+0.64X3X4 \end{array}$$

$$\begin{array}{c} \text{YColor}=3.28X1+4.83X2+2.90X3+4.41X4\\ \;+2.23X1X2-2.75X1X3-8.77X1X4\\ \;+2.39X2X3-4.83X2X4+2.61X3X4 \end{array}$$

$$\begin{array}{c} \text{YOverall}\;\mathrm{acc}.=2.79X1+0.72X2-0.16X3+2.11X4+5.15X1X2\\ \;-0.09\;X1X3-8.71X1X4+0.98X2X3-6.60X2X4\\ \;+2.39X3X4 \end{array}$$

**FIGURE 2:**
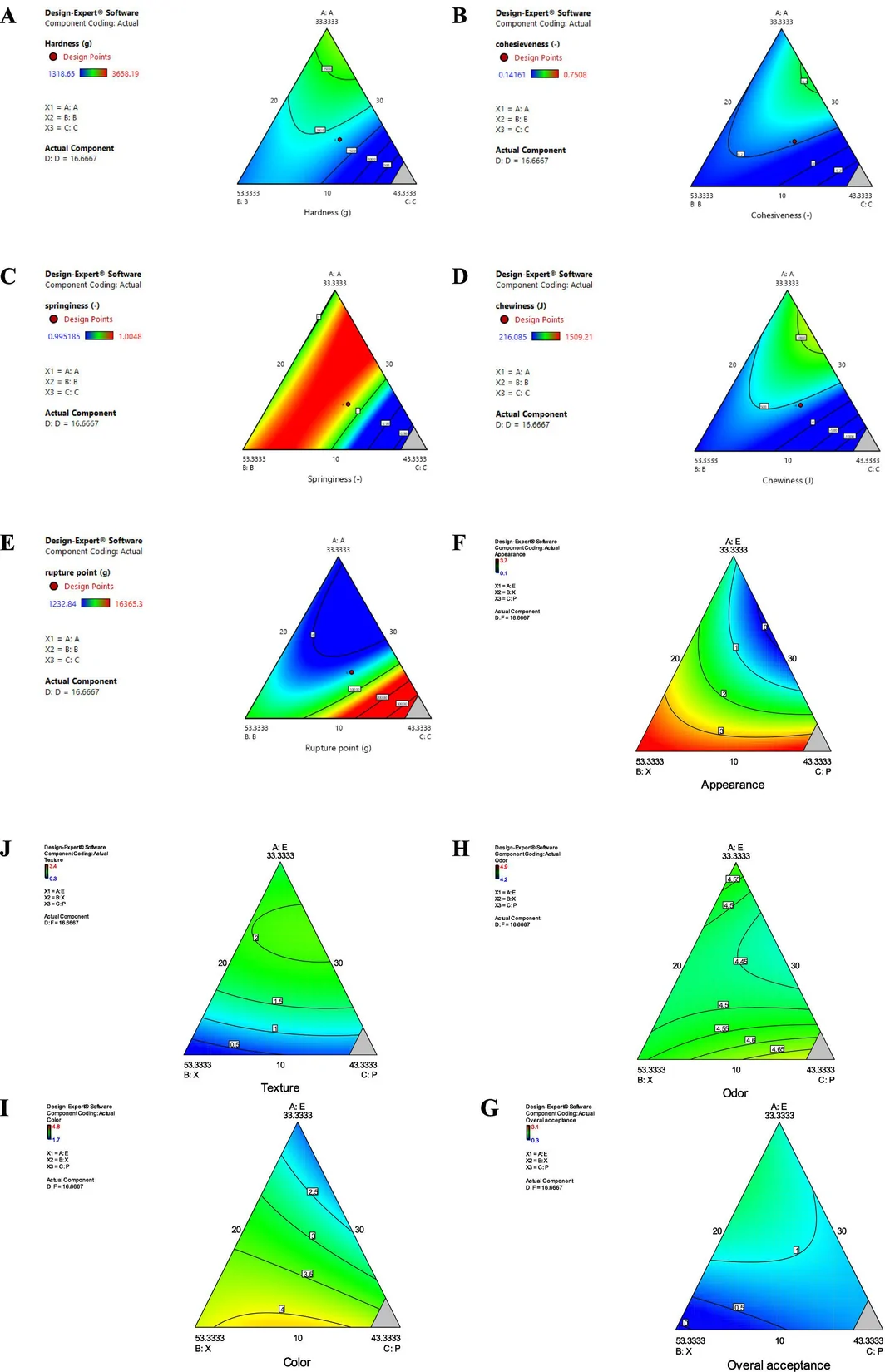
2D contour plots showing the effect of PPLA/Saqqez gum elastomer (*X1*), xylitol (*X2*), and emulsifier (*X3*), at different levels on (A) hardness, (B) cohesiveness (C) springiness, (D) chewiness, (E) rupture point, (F) appearance, (J) texture (H) odor, (I) color, and (G) overall acceptance of the chewing gum samples while maintaining the filler (*X4*) constant (16.6%) at mid‐point. Regions that do not fit the optimization criteria are shaded in gray.

In addition to the coefficients, the 2‐dimensional contour plots shown in Figure 2 depict the effects of *X1*, *X2*, and *X3* on the responses, while keeping the remaining variable (*X4*) constant at its mid‐point. These triangular contour plots provide valuable insights into the relationship between components with varying mixture proportions. Vertices A, B, and C represent pure *X1*, *X2*, and *X3* components, respectively. The connecting axes represent binary mixtures, and contour lines within the triangle denote points with equal response values. The color gradient or shading reflects different response levels. Darker areas indicate higher values, while lighter areas represent lower values. Close contour lines together suggest a strong effect of variables on the response, while widely spaced lines indicate a weaker effect.

*X1*, representing the PPLA/Saqqez gum blend, notably enhances all attributes, especially hardness, chewiness, and rupture point, while minimally affecting cohesiveness. The results indicate that increasing its proportion leads to greater hardness and improved chewability. Al Hagbani et al. (2018) revealed that elastomers exhibit elasticity due to their long polymer chains reconfiguring under applied stress. When the total elastomer content is below 10%, the gum base loses its desired texture and cohesiveness. However, at amounts exceeding 30%, the gums become excessively hard and rubbery. Therefore, determining the optimal elastomer level is crucial for achieving the desired properties (Al Hagbani et al. 2018). It is generally expected that increasing the solids content in chewing gum will reduce its elasticity and increase its stiffness. Therefore, texturizing agents help harden thermoplastic polymers (Al Hagbani et al. 2018), while filler and xylitol also contribute (Kaveh et al. 2023). On the other hand, producing softer chewing gum or similar products carries the risk of structural deformations during transportation, storage, and sales. Therefore, it is crucial to manufacture chewing gum with an optimal hardness to ensure consumer appeal and align with sales strategies. Additionally, the hardness affects aroma release, a key quality parameter for chewing gum (Palabiyik et al. 2020).

*X2* (xylitol) generally has a positive effect on all attributes. The results indicate that xylitol, similar to the elastomer, enhances hardness and chewiness. This aligns with previous studies that highlighted its texturizing properties (Kaveh et al. 2023). According to Muscat et al. (2012), sometimes xylitol acts as a plasticizer. This behavior may be attributed to its relatively larger molecular size and stronger hydrogen bonding with other molecules (Muscat et al. 2012). In confirmation of this study, xylitol resulted in a significant increase in the rupture point. The findings also reveal that xylitol positively influences all sensory attributes, which likely contributes to the consumer's acceptance of sweet products (Winkelhausen et al. 2007).

*X3* (glycerin) shows mixed effects, notably reducing chewiness, cohesiveness, and hardness. Chewiness is one important parameter for achieving excellent properties in chewing gum. Adjusting the chewiness of chewing gum is crucial for enhancing sensory acceptance. A lower chewiness value is beneficial as it reduces the energy required during chewing (Santos et al. 2014), highlighting the dominant role of this component. Glycerin, with its plasticizing function, also reduces hardness (Kaveh et al. 2023), consistent with our results. Lees and Jackson (1973) noted that emulsifiers like lecithin and glycerin function as plasticizers, enhancing the softness of products (Lees and Jackson 1973). Hence, it can lead to a soft texture that agrees with the negative impact of glycerin on the texture sensory attribute and subsequently causes a negative effect on overall acceptance, as shown in the results. Another critical parameter is cohesiveness, which refers to the extent a material can be stretched or deformed before breaking (Szczesniak 2002). Findings indicate that an increase in *X3* enhances cohesiveness. Tarade and colleagues suggested that plasticizers, such as glycerol, regulate the cohesiveness of chewing gum (Tarade Vijay et al. 2016). Attar et al. (2018) proposed that glycerin acts as a plasticizer, leading to water retention in the product. Consequently, increasing glycerin concentration can alter the product's moisture due to the glycerol‐gum interactions (Attar et al. 2018). Sometimes glycerin, acting as an elastomer solvent, connects elastomers with other materials, maintaining the balance between the elastic and plastic properties of the gum base (Khairnar et al. 2016).

*X4* (calcium carbonate) mostly has positive impacts, significantly affecting hardness. Generally, increased filler content leads to greater hardness, confirming calcium carbonate's dominant texturizing role as a filler (Li et al. 1985). Also, it exhibits negative effects on rupture point. Calcium carbonate can diminish the elasticity of materials. When incorporated into polymers, it typically enhances stiffness and tensile strength but reduces elasticity and impact resistance (Salfitra and Putra 2023).

In terms of interactions between components (*X1X2*, *X1X3*, *X1X4*, *X2X3*, *X2X4*, and *X3X4*), interpreting the coefficients presented in Table S3 can be somewhat complicated. These coefficients represent the estimated impact of two (L‐pseudo) components interacting with each other while keeping all other components constant.

The interaction effects are as follows: *X1X2* negatively impact most attributes, except cohesiveness, texture, color, and overall acceptance. *X1X3* has a significant positive impact on several attributes but a negative effect on rupture point and all sensory attributes, except for texture. *X1X4* generally has negative impacts on all attributes. Both *X1* and *X4* have a significant positive effect on hardness, but the interaction between them (*X1X4*) has the opposite effect. *X2X3* has just a negative impact on rupture point. *X2X4* has positive effects only on springiness, rupture point, and appearance. *X3X4* has negative effects only on rupture point and appearance.

The 2D contour plot in Figure 2, panel B, illustrates the positive effect of *X1X3* interaction on cohesiveness, indicated by the greenish color near the *X1X3* axis. While other interactions between *X1*, *X2*, and *X3* also have positive effects, this area is most dominant. Similarly, panel D demonstrates the positive effect of *X1X3* on chewiness, consistent with their coefficients. Panel E shows that increasing *X3* leads to a higher rupture point, aligning with the coefficients in Table S3. Panel F shows how increases in the value of *X2* and *X3* lead to a decrease in appearance.

### 3.3 Optimization of the Chewing Gum Components

The objective of the optimization process during the development of chewing gum formulations is to determine the optimal levels for each variable that ensures the creation of a robust product with high‐quality characteristics. To achieve this objective, target responses were assigned to all dependent variables. Subsequently, an 81.7% desirability was considered using software. The composition of the optimized formulation is as follows: 39.40% PPLA/Saqqez gum blend as the elastomer of the gum base, 30.59% xylitol as the bulking sweetener, 20% glycerin as the emulsifier, and 10% calcium carbonate as the filler. Additionally, its predicted response for hardness, cohesiveness, springiness, chewiness, rupture point, appearance, texture, odor, color and overall acceptance are 3170 g, 0.372, 1.003, 1241.5, 6530.8 g, 3.630, 3.400, 4.681, 3.356, 3.947, respectively. The validity of the predicted models was confirmed. Approximately, all of these values fall within the target range of responses that are mentioned in Table 1.

### 3.4 Characterization Results

In this step, a new formulation was created based on optimized levels of components (Op chewing gum) and compared with two commercial samples, SaC and SyC chewing gum, in the following tests.

#### 3.4.1 Moisture and Ash Content

Table 5 indicates moisture and ash contents of the samples. The total ash content of SaC chewing gum (0.84% ± 0.23%) was markedly lower than both SyC chewing gum (9.61% ± 0.91%) and Op chewing gum (10.52% ± 0.26%). In contrast, the SyC sample showed an ash content close to that of the Op chewing gum. These differences were statistically significant. The higher ash content of the Op sample indicates a greater total inorganic mineral fraction. The similarity between Op and SyC may be related to the presence of filler in both formulations, unlike SaC. Additionally, it has been reported that the ash contents of Saqqez gum were 0.29% (Mohammadi et al. 2019).

**TABLE 5:** Moisture, ash content, and thermal parameters of the samples.

Samples	Moisture (%)	Ash (%)	Tg (°C)	Tm (°C)
Op chewing gum	0.50 ± 0.11^c^	10.52 ± 0.26^a^	53.6 ± 1.2^a^	146.3 ± 2.5^a^
SaC chewing gum	1.33 ± 0.67^b^	0.84 ± 0.23^b^	54.4 ± 1.8^a^	141.3 ± 3.1^b^
SyC chewing gum	2.50 ± 0.32^a^	9.61 ± 0.91^a^	43.2 ± 0.9^b^	91.2 ± 4.0^c^

*Note:* Values are reported as mean ± standard deviation (SD). Lowercase letters indicate statistically significant differences (*p* < 0.05) among sample groups for each parameter.

Based on the findings, the Op chewing gum exhibited the lowest moisture content, while SaC chewing gum had a higher moisture content, and SyC chewing gum had the highest. This indicates that the Op chewing gum was quite a dry sample, whereas the SyC chewing gum was the moistest among the samples (Table 5).

#### 3.4.2 Thermal Properties

The thermal properties of the optimized (Op) chewing gum were assessed using Differential Scanning Calorimetry (DSC), which is pivotal for evaluating glass transitions (Tg) and melting temperatures (Tm) within chewing gum systems. The DSC is useful to assessing the glass transitions (Tg) of chewing gums and identifying any change in the controlling processes of the samples (Welti‐Chanes et al. 2008). Table 5 displays the Tg and Tm of all chewing gum samples. The Op chewing gum exhibited a Tg peak at 53.6°C ± 1.2°C, which indicates a crucial transition point for texture and chewability. In comparison, the SaC and SyC chewing gums showed Tg values close to the Op sample but significantly diverged in their melting endotherms peaks at 141.3°C ± 3.1°C, and 91.2°C ± 4.0°C, respectively. Understanding these thermal transitions is essential as they directly influence sensory and textural parameters. The Tg serves as an indicator of the firmness at oral temperatures, affecting chewability; higher Tg values typically correlate with a more rigid structure, which can contribute to a firmer chewing sensation. In contrast, the relatively lower Tg values in SaC and SyC suggest that these gums may possess a softer, more pliable texture, enhancing chewability and potentially leading to a more enjoyable sensory experience. Additionally, DSC results show that the Tm is integral to understanding the gum's behavior during chewing. The melting temperature provides insights into the elasticity and stickiness of the gum. Specifically, a higher Tm value, such as that of SaC, indicates greater resistance to thermal softening, which might translate to a firmer texture and enhanced chew resistance, potentially contributing to a prolonged chewing experience without significant softening. Conversely, the lower Tm of SyC could imply a quicker softening under oral conditions, which may enhance the initial chewability but compromise the gum's structural integrity during prolonged chewing. The variations in Tg and Tm highlight the structural and compositional differences among the samples. The miscibility of pure polymers, polymer blends, copolymers, and polymer‐based composites is determined by their Tg, which vary with composition and significantly impact their properties (Brostow et al. 2008). As established by previous research, Qian et al. (2010) observed that the distinct Tg of an amorphous mixture represents the weighted average Tg of its individual components, indicating molecular‐level homogeneity (Qian et al. 2010). For instance, the observed structural rigidity of the optimized formulation, manifesting from its higher Tg, points to a balance that supports chewability while ensuring that the gum remains elastic enough to avoid excessive breakage. Additionally, shifts in Tg values denote changes in the softness and elasticity of samples (Mohammadi et al. 2019). Thus, variations in Tg reflect differences in the structural composition of chewing gums. Higher Tg values correlate with more rigid structures, whereas lower Tg values suggest softer, more flexible structures, providing valuable insights for formulating chewing gums with specific textural properties. Previous studies have demonstrated that the endothermic melting temperature (Tm) is influenced by the molecular motion of polymer chains and the degradation of their molecular structure. This phenomenon can be attributed to the evaporation of moisture from the sample or the degradation of the softener, both of which significantly affect the properties and applications of the materials (Ghaderi et al. 2021; Ibrahim et al. 2013). The Tm values correspond to the moisture content of the samples (Mohammadi et al. 2019), with higher Tm values observed in samples with lower moisture levels, consistent with the optimal sample's characteristics. Ultimately, these detailed interpretations provide valuable insights into how the optimized formulation may perform in real‐world consumer experiences, emphasizing that the optimized gum's higher Tg and Tm values contribute positively to the intended chewing characteristics, aligning them with desired sensory attributes of firmness, chewability, and elasticity.

#### 3.4.3 Morphological Properties

**FIGURE 3:**
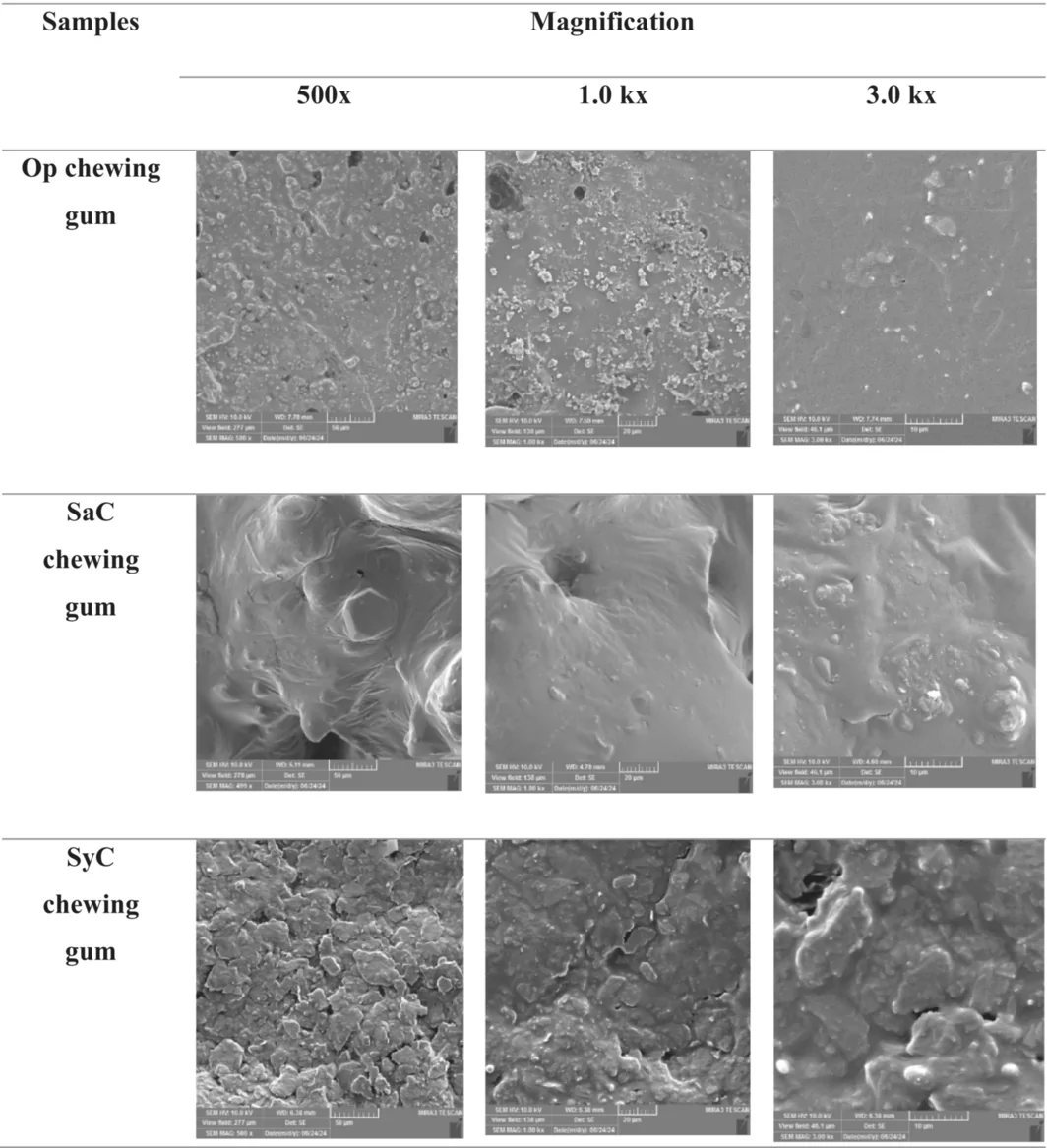
SEM micrographs of Op chewing gum, SaC, and SyC chewing gum at the indicated magnification.

FESEM was employed to examine the fracture surface morphologies, enabling the analysis of particle dispersion, sample differences, and the miscibility between matrices and materials (Kaveh et al. 2024). Figure 3 illustrates the samples at three different magnifications. At 500× magnification, the Op chewing gum sample shows a relatively smooth surface with some scattered particles. Immiscible polymer blends typically display various morphologies, such as sea‐island structures, influenced by the polymer compound ratio, interfacial adhesion, and processing conditions (Wei et al. 2021). The PPLA/Saqqez gum polymer blend in the Op chewing gum sample exhibits sea‐island structures, with two phases forming discrete spherical domains within a surrounding matrix (Hu et al. 2018), more evident at 1.0 k× and 3.0 k× magnifications. The SaC chewing gum images at 500× magnification reveal a highly textured and porous surface, becoming more pronounced at higher magnifications, including mixture homogeneity without sea‐island structures. The SyC chewing gum image at 500× magnification shows a rough and cracked surface, with the clustered granular formations becoming more visible at 3.0 k×, indicating a less uniform surface compared to the other samples. Comparing the two Op and SyC samples reveals that the Op sample exhibits more porous and aerated spaces. In contrast, the latter sample is more compact and freer of porosity. These differences can be attributed to the distinct production processes and equipment used for each of them.

#### 3.4.4 Biodegradability Test

**FIGURE 4:**
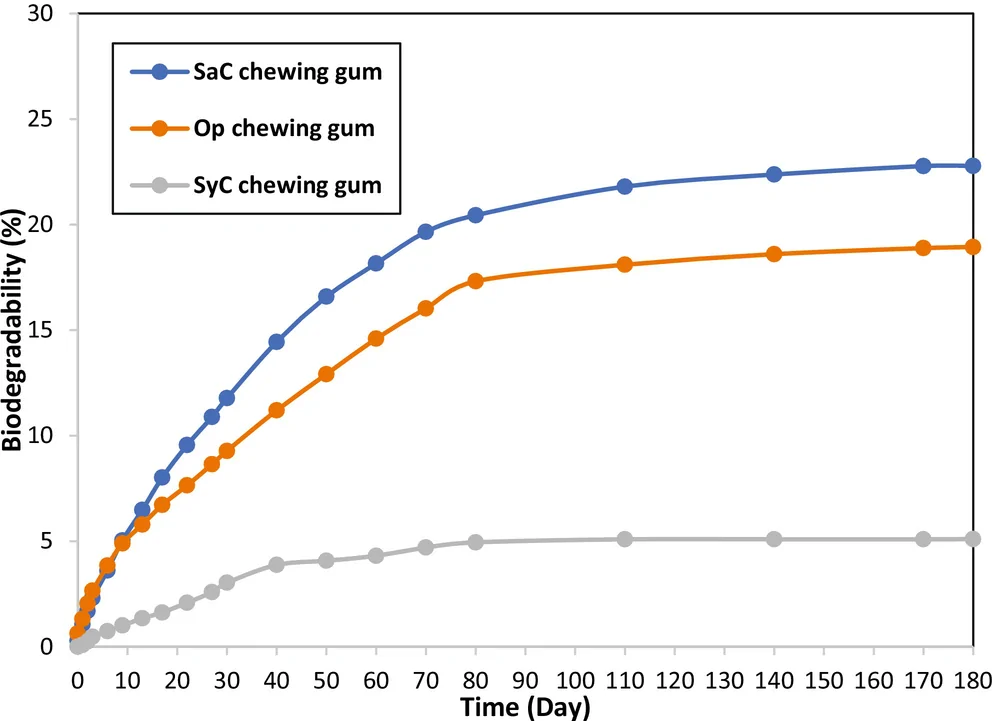
Biodegradability of Op chewing gum and commercial samples under aerobic conditions at 25°C during 6 months.

The biodegradability of the Op chewing gum, along with SaC and SyC chewing gums, is illustrated in Figure 4. By the 40th day of analysis, the biodegradability of the Op chewing gum surpassed 11%, while SaC and SyC chewing gum reached approximately 14.44% and 3.03%, respectively. After 6 months, the Op chewing gum exhibited an 18.94% biodegradability, ranking second compared to SaC chewing gum at 22.78%. The SyC chewing gum showed the lowest biodegradability at 5.12%, attributed to its non‐biodegradable components in the gum base. Saberi et al. (2018) also reported that the biodegradability of their Saqqez bio‐chewing gum after a 20‐weeks period was 14.34%, which was very similar to the results of SaC chewing gum in our study (Saberi et al. 2018). Consistent with these observations, previous research has shown that incorporating natural plant‐derived components into polymer‐based materials can enhance their breakdown in soil, thereby supporting environmental sustainability (Alshehri et al. 2024).

#### 3.4.5 Textural Properties

Table 6A presents the findings from the TPA. Hardness, which measures the peak force required to first compress the chewing gum (Mardani et al. 2019), is highest in Op chewing gum, followed by SaC, and then SyC chewing gum. The elastomeric component of SyC chewing gum, typically polyvinyl acetate, serves as a texture modifier, enhancing cohesiveness and strength, which is crucial for achieving desirable texture and chewing properties (Kaveh et al. 2023). Adhesiveness, which measures how much the gum sticks to surfaces (Mardani et al. 2019), is highest in SaC chewing gum. In contrast, the Op and SyC chewing gums had relatively low values, indicating they were less sticky and easier to chew without sticking to teeth. Cohesiveness, which relates to the degree to which a substance can be deformed before breaking (Rosenthal and Thompson 2021), is highest in Op chewing gum, followed by SaC, and SyC chewing gum. Springiness, measuring how well the gum returns to its original shape after being compressed (Di Monaco et al. 2008), is relatively similar across all three samples, with Op chewing gum being slightly springier. Chewiness, which combines hardness, cohesiveness, and springiness to measure the energy required to chew the chewing gum (Kiumarsi et al. 2019), is highest in Op chewing gum, followed by SaC, and then SyC chewing gum. In summary, Op chewing gum is the hardest, cohesive, springy, and chewy compared to the commercial samples. SaC chewing gum is moderately hard, most adhesive, and cohesive, while SyC chewing gum is the least hard, cohesive, and chewy.

**TABLE 6:** (A) TPA, and (B) sensory attributes intensity results of Op, SaC and SyC chewing gum samples.

	Samples	Hardness (g)	Adhesiveness (g.s)	Cohesiveness (−)	Springiness (−)	Chewiness (J)	
A	Op chewing gum	3168.18 ± 388.95^a^	−58.51 ± 7.20^b^	0.37 ± 0.07^a^	1.04 ± 0.01^a^	1219.11 ± 242.32^a^	
	SaC chewing gum	2710.41 ± 772.16^b^	−170.29 ± 3.51^a^	0.23 ± 0.02^b^	0.97 ± 0.06^b^	604.69 ± 228.17^b^	
	SyC chewing gum	1932.54 ± 38.18^c^	−41.81 ± 1.77^c^	0.14 ± 0.06^c^	0.98 ± 0.03^b^	265.14 ± 52.63^c^	
	**Samples**	**Hardness**	**Moistness**	**Stickiness**	**Cohesiveness**	**Chewiness**	**Sandiness**
B	Op chewing gum	3.21 ± 0.04^a^	1.42 ± 0.05^b^	1.00 ± 0.08^a^	2.36 ± 0.04^c^	3.26 ± 0.00^b^	0.05 ± 0.02^a^
	SaC chewing gum	3.47 ± 0.06^a^	1.21 ± 0.09^b^	0.59 ± 0.06^b^	3.52 ± 0.01^b^	2.84 ± 0.06^c^	0.01 ± 0.01^b^
	SyC chewing gum	2.052 ± 0.06^b^	3.004 ± 0.01^a^	0.105 ± 0.10^c^	4.473 ± 0.03^a^	4.052 ± 0.01^a^	0.052 ± 0.04^a^

*Note:* Values are expressed as mean ± standard deviation (*n* = 3). Different superscript letters within the same column (for each experiment) indicate significant differences among treatments (*p* < 0.05).

#### 3.4.6 Quantitative Sensory Descriptive Analysis

**FIGURE 5:**
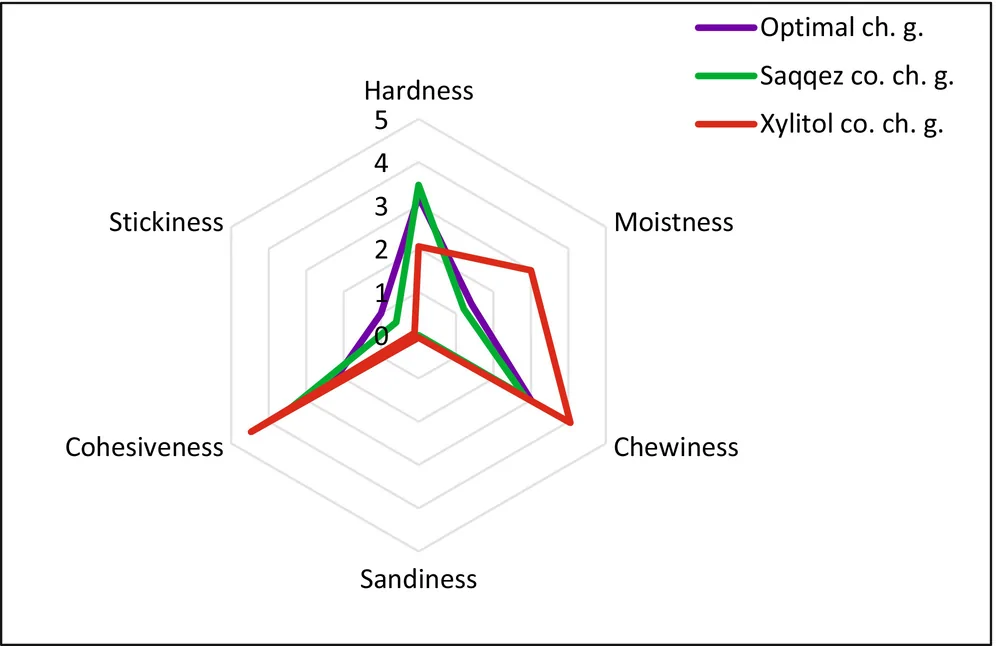
Sensory profile of chewing gum samples using spider web diagram.

Quantitative sensory descriptive analysis is a widely used method for descriptive testing. It trains participants to evaluate specific attributes, leading to a comprehensive product description. A key benefit of this test is its accuracy, as it uses trained participants. Additionally, it is effective in identifying highly sensitive attributes (Kiumarsi et al. 2020). The test results, analyzed using ANOVA, revealed differences between the samples as shown in Table 6B. The intensity of hardness, moistness, and sandiness were relatively similar in both Op and SaC chewing gum, placing them in the same group based on the significance level. The SyC chewing gum exhibits higher intensity levels of moistness, cohesiveness, and chewiness compared to other samples. Figure 5 visually represents the evaluation of the samples using a spider web plot (or radar chart), focusing on six sensory attributes: hardness, moistness, cohesiveness, stickiness, chewiness, and sandiness. The spider web plot illustrated the intensity comparison of these sensory attributes among the samples. The Op sample exhibited sensory attributes most similar to the SaC sample, indicating potential competition between them. Both the Op and SaC samples were perceived as hard and cohesive, with relatively low levels of moistness, stickiness, and sandiness compared to the SyC sample. SyC chewing gum showed high chewiness and cohesiveness, along with moderate hardness and moistness.

## 4 Conclusion

The impact of common ingredients in chewing gum formulations on its textural and sensory properties has not been systematically studied for PPLA/Saqqez gum elastomer‐based biodegradable gum bases using D‐optimal mixture design. This study, using experimental mixture design, found that all attributes are significantly influenced by the quadratic model. The PPLA/Saqqez gum blend and xylitol notably enhance all sensory attributes, significantly increasing hardness, cohesiveness, chewiness, and rupture point. The interaction between PPLA/Saqqez gum blend and xylitol significantly increased the overall acceptance of the chewing gum. An 81.7% desirability plot was generated, showing that approximately all attribute values fall within the target range of responses. Comparing the Op chewing gum with two commercial samples revealed that the optimal chewing gum is the hardest, most cohesive, springy, and chewy, with higher total inorganic mineral content. In conclusion, this investigation suggests that focusing on the production of innovative, biodegradable products is a promising path for the food industry. These products not only match commercial equivalents in some aspects but also have better performance in certain areas, deserving further studies to address any shortcomings.

## Author Contributions

**Mona Kaveh:** investigation, writing – original draft, methodology, software. **Samira Yeganehzad:** conceptualization, writing – review and editing, methodology, supervision, funding acquisition, validation, resources. **Mohammad Ali Hesarinejad:** conceptualization, funding acquisition, methodology, validation, writing – review and editing, supervision, software, resources.

## Funding

This work was supported by Iran National Science Foundation, 4036280.

## Ethics Statement

This study received approval from the Research Institutes of Food Science and Technology (RIFST).

## Consent

Before the test, participants received a consent letter explaining all details about the study's purpose and, if they agreed, signed it.

## Conflicts of Interest

The authors declare no conflicts of interest.

## Supporting information


**Table S1:** Suggested models for the response variables in a mixture design experiment.
**Table S2:** Analysis of Variance (ANOVA) for the responses.
**Table S3:** Polynomial models for the responses based on L‐Pseudo components.

## Data Availability

All data generated or analyzed during this study are included in this published article.
