# Supplementary material for: Development of Biodegradable Chewing Gum Using Plasticized Poly(Lactic Acid)/Pistacia atlantica Gum Blend: A Novel Gum Base Elastomer for Sustainable Formulation
Source: Food Sci Nutr. 2026 Sep 7;14(9):e72322. doi: 10.1002/fsn3.72322 (PMC13550758; doi:10.1002/fsn3.72322)
Supplement: Supplementary file 1 [file FSN3-14-e72322-s001.docx]

Appendix:

Supplementary Tables:

**Table S1. Suggested models for the response variables in a mixture design experiment**

| Attributes | Source | Std. Dev. | R^2^ | Adjusted R^2^ | Predicted R^2^ | PRESS |  |
| --- | --- | --- | --- | --- | --- | --- | --- |
| Hardness | **Linear** | 209.20 | 0.9154 | 0.8985 | 0.8584 | 1.099E+06 | Suggested |
|  | **Quadratic** | 45.41 | 0.9741 | 0.9290 | 0.6967 | 9.346E+06 | Suggested |
|  | **Special Cubic** | 0.000 | 1.0000 | 1.0000 |  | + | Aliased |
| Cohesiveness | **Linear** | 0.1986 | 0.2522 | 0.1026 | -0.2586 | 0.9964 |  |
|  | **Quadratic** | 0.0637 | 0.9538 | 0.9075 | -22.2831 | 18.41 | Suggested |
|  | **Special Cubic** | 0.000 | 1.0000 | 1.0000 |  | + | Aliased |
| Springiness | **Linear** | 0.0035 | 0.1084 | -0.0699 | -0.3943 | 0.0003 |  |
|  | **Quadratic** | 0.0023 | 0.7728 | 0.8978 | 0.6169 | 0.0242 | Suggested |
|  | **Special Cubic** | 0.000 | 1.0000 | 1.0000 |  | + | Aliased |
| Chewiness | **Linear** | 400.07 | 0.7330 | 0.4163 | 0.1786 | 1.188E+009 | Suggested |
|  | **Quadratic** | 158.36 | 0.9263 | 0.8984 | -24.5810 | 1.137E+010 | Suggested |
|  | **Special Cubic** | 0.000 | 1.0000 | 1.0000 |  | + | Aliased |
| Rupture point | **Linear** | 2.057 | 0.6771 | 0.3464 | 0.0740 | 1.059E+009 | Suggested |
|  | **Quadratic** | 0.323 | 0.9019 | 0.7547 | 0.7745 | 6.968E+010 | Suggested |
|  | **Special Cubic** | 0.000 | 1.0000 | 1.0000 |  | + | Aliased |
| Appearance | **Linear** | 1.540 | 0.2799 | 0.0998 | 0.2563 | 49.40 |  |
|  | **Quadratic** | 0.400 | 0.9761 | 0.9402 | 1.8132 | 75.228 | Suggested |
|  | **Special Cubic** | 0.000 | 1.0000 | 1.0000 |  | + | Aliased |
| Texture | **Linear** | 1.020 | 0.7666 | 0.4583 | 0.3094 | 19.73 | Suggested |
|  | **Quadratic** | 0.230 | 0.9892 | 0.9731 | 0.7600 | 24.577 | Suggested |
|  | **Special Cubic** | 0.000 | 1.0000 | 1.0000 |  | + | Aliased |
| Odor | **Linear** | 0.170 | 0.6148 | 0.5185 | -0.3578 | 0.56 | Suggested |
|  | **Quadratic** | 0.096 | 0.9367 | 0.8417 | 1.9621 | 4.426 | Suggested |
|  | **Special Cubic** | 0.000 | 1.0000 | 1.0000 |  | + | Aliased |
| Color | **Linear** | 0.850 | 0.7426 | 0.4283 | 0.2606 | 13.90 | Suggested |
|  | **Quadratic** | 0.150 | 0.9925 | 0.9811 | 0.5031 | 11.337 | Suggested |
|  | **Special Cubic** | 0.000 | 1.0000 | 1.0000 |  | + | Aliased |
| Overall acceptance | **Linear** | 0.900 | 0.3164 | 0.1455 | 0.0600 | 15.07 |  |
|  | **Quadratic** | 0.100 | 0.9954 | 0.9885 | 0.8763 | 52.28 | Suggested |
|  | **Special Cubic** | 0.000 | 1.0000 | 1.0000 |  | + | Aliased |

+ Case(s) with leverage of 1.0000: PRESS statistic not defined

**Table S2. Analysis of Variance (ANOVA) for the responses**

| ANOVA for the quadratic model for Hardness | | | | | | |
| --- | --- | --- | --- | --- | --- | --- |
| Source | **Sum of Square** | **df** | **Mean Square** | **F value** | **p-value** |  |
| Model | 7.742E+06 | 9 | 8.601E+05 | 11.27 | 0.0040 | Significant |
| Residual | 1055.69 | 9 | 201.85 |  |  |  |
| Lack of fit | 1855.69 | 1 | 1855.69 |  |  |  |
| Pure error | 0.000 | 8 | 0.000 |  |  |  |
| Corrected total | 7.760 | 18 |  |  |  |  |
| ANOVA for the quadratic model for Cohesiveness | | | | | | |
| Source | **Sum of Square** | **df** | **Mean Square** | **F value** | **p-value** |  |
| Model | 0.7543 | 9 | 0.0838 | 20.63 | 0.0027 | Significant |
| Residual | 0.0366 | 9 | 0.0041 |  |  |  |
| Lack of fit | 0.366 | 1 | 0.366 |  |  |  |
| Pure error | 0.000 | 8 | 0.000 |  |  |  |
| Corrected total | 0.7909 | 18 |  |  |  |  |
| ANOVA for the quadratic model for Springiness | | | | | | |
| Source | **Sum of Square** | **df** | **Mean Square** | **F value** | **p-value** |  |
| Model | 0.0002 | 9 | 0.0000 | 3.40 | 0.0412 | Significant |
| Residual | 3.913E-006 | 9 | 8.272E-007 |  |  |  |
| Lack of fit | 4.963 | 1 | 4.963 |  |  |  |
| Pure error | 0.000 | 8 | 0.000 |  |  |  |
| Corrected total | 1.214E-004 | 18 |  |  |  |  |
| ANOVA for the quadratic model for Chewiness | | | | | | |
| Source | **Sum of Square** | **df** | **Mean Square** | **F value** | **p-value** |  |
| Model | 4.218 | 9 | 4.687 | 18.69 | 0.0008 | Significant |
| Residual | 2.157 | 9 | 277.56 |  |  |  |
| Lack of fit | 2.257 | 1 | 2.257 |  |  |  |
| Pure error | 0.000 | 8 | 0.000 |  |  |  |
| Corrected total | 4.446 | 18 |  |  |  |  |
| ANOVA for the quadratic model for Rupture point | | | | | | |
| Source | **Sum of Square** | **df** | **Mean Square** | **F value** | **p-value** |  |
| Model | 6.088 | 9 | 6.764 | 8.83 | 0.0017 | Significant |
| Residual | 6.006 | 9 | 7.662 |  |  |  |
| Lack of fit | 6.896 | 1 | 6.896 |  |  |  |
| Pure error | 0.000 | 8 | 0.000 |  |  |  |
| Corrected total | 6.778 | 18 |  |  |  |  |
| ANOVA for the quadratic model for Appearance | | | | | | |
| Source | **Sum of Square** | **df** | **Mean Square** | **F value** | **p-value** |  |
| Model | 38.38 | 9 | 4.26 | 27.20 | 0.0003 | Significant |
| Residual | 0.94 | 9 | 0.16 |  |  |  |
| Lack of fit | 0.94 | 1 | 0.94 |  |  |  |
| Pure error | 0.000 | 8 | 0.000 |  |  |  |
| Corrected total | 39.32 | 18 |  |  |  |  |
| ANOVA for the quadratic model for Texture | | | | | | |
| Source | **Sum of Square** | **df** | **Mean Square** | **F value** | **p-value** |  |
| Model | 28.27 | 9 | 3.14 | 61.32 | <0.0001 | Significant |
| Residual | 0.31 | 9 | 0.051 |  |  |  |
| Lack of fit | 0.31 | 1 | 0.31 |  |  |  |
| Pure error | 0.000 | 8 | 0.000 |  |  |  |
| Corrected total | 28.58 | 18 |  |  |  |  |
| ANOVA for the quadratic model for Odor | | | | | | |
| Source | **Sum of Square** | **df** | **Mean Square** | **F value** | **p-value** |  |
| Model | 0.82 | 9 | 0.091 | 9.86 | 0.0057 | Significant |
| Residual | 0.055 | 9 | 9.226E-003 |  |  |  |
| Lack of fit | 0.055 | 1 | 0.055 |  |  |  |
| Pure error | 0.000 | 8 | 0.000 |  |  |  |
| Corrected total | 0.87 | 18 |  |  |  |  |
| ANOVA for the quadratic model for Color | | | | | | |
| Source | **Sum of Square** | **df** | **Mean Square** | **F value** | **p-value** |  |
| Model | 18.66 | 9 | 2.07 | 87.72 | <0.0001 | Significant |
| Residual | 0.14 | 9 | 0.024 |  |  |  |
| Lack of fit | 0.14 | 1 | 0.14 |  |  |  |
| Pure error | 0.000 | 8 | 0.000 |  |  |  |
| Corrected total | 18.80 | 18 |  |  |  |  |
| ANOVA for the quadratic model for Overall acceptance | | | | | | |
| Source | **Sum of Square** | **df** | **Mean Square** | **F value** | **p-value** |  |
| Model | 14.15 | 9 | 1.57 | 144.34 | <0.0001 | Significant |
| Residual | 0.065 | 9 | 0.011 |  |  |  |
| Lack of fit | 0.065 | 1 | 0.065 |  |  |  |
| Pure error | 0.000 | 8 | 0.000 |  |  |  |
| Corrected total | 14.22 | 18 |  |  |  |  |

**Table S3. Polynomial models for the responses based on L-Pseudo components**

| Coefficient | Hardness | Cohesiveness | Springiness | Chewiness | Rupture p. | Appearance | Texture | Odor | Color | Overall. acceptance |
| --- | --- | --- | --- | --- | --- | --- | --- | --- | --- | --- |
| X1 | +3642.44 | +0.39 | +1.00 | +1421.19 | +7488.24 | +3.55 | +3.33 | +4.59 | +3.28 | +2.79 |
| X2 | +1655.30 | +0.24 | +1.00 | +431.59 | +2770.63 | +3.67 | +0.26 | +4.82 | +4.83 | +0.72 |
| X3 | -3277.65 | -1.26 | +0.9238 | -4472.36 | +92529.28 | +5.18 | -0.13 | +4.51 | +2.90 | -0.16 |
| X4 | +1595.42 | +0.76 | +0.9983 | +1223.43 | -13786.76 | +3.43 | +1.68 | +4.91 | +4.41 | +2.11 |
| X1X2 | -1623.63 | +0.09 | -0.0057 | -644.88 | -16800.92 | -1.35 | +6.95 | -0.63 | +2.23 | +5.15 |
| X1X3 | +6799.30 | +3.67 | +0.1388 | +9965.71 | -1.788E+05 | -17.62 | +6.52 | -1.60 | -2.75 | -0.090 |
| X1X4 | -2399.64 | -1.56 | -0.0138 | -3783.14 | -37466.81 | -13.23 | -8.15 | -0.30 | -8.77 | -8.71 |
| X2X3 | +7118.16 | +1.52 | +0.1092 | +5697.29 | -94491.03 | +3.58 | +1.34 | +0.14 | +2.39 | +0.98 |
| X2X4 | -544.98 | -1.27 | +0.0263 | -2293.02 | +24576.23 | +2.11 | -2.99 | -1.74 | -4.83 | -6.60 |
| X3X4 | +7808.67 | +1.02 | +0.1303 | +5630.54 | -1.410E+05 | -3.45 | +1.41 | +0.64 | +2.61 | +2.39 |

X1: Elastomer/ X2: Xylitol/ X3: Emulsifier/ X4: Filler
